# RBP-J is not required for granule neuron progenitor development and medulloblastoma initiated by Hedgehog pathway activation in the external germinal layer

**DOI:** 10.1186/1749-8104-5-27

**Published:** 2010-10-15

**Authors:** Elaine Julian, Andrew R Hallahan, Brandon J Wainwright

**Affiliations:** 1 Institute for Molecular Bioscience, University of Queensland, Brisbane, Australia; 2 Department of Paediatrics and Child Health, University of Queensland, Brisbane, Australia

## Abstract

**Background:**

The Notch signalling pathway plays crucial roles in neural development, functioning by preventing premature differentiation and promotion of glial cell fates. In the developing cerebellum Notch pathway components are expressed in granule neuron progenitors of the external germinal layer (EGL) but the precise function of Notch in these cells is unclear. The Hedgehog pathway is also crucial in cerebellar development, mainly via control of the cell cycle, and persistent activation of the pathways leads to the cerebellar tumour medulloblastoma. Interactions between Hedgehog and Notch have been reported in normal brain development as well as in Hedgehog pathway induced medulloblastoma but the molecular details of this interaction are not known and we investigate here the role of Notch signalling in the development of the EGL and the intersection between the two pathways in cerebellar granule neuron progenitors and in medulloblastoma.

**Results:**

RBP-J is the major downstream effector of all four mammalian Notch receptors and the RBP-J conditional mouse facilitates inactivation of canonical Notch signals. Patched1 is a negative regulator of Hedgehog signalling and the Patched1 conditional mouse is widely used to activate Hedgehog signalling via Patched1 deletion in specific cell types. The conditional mouse lines were crossed with a Math1-Cre line to delete the two genes in granule neuron progenitors from embryonic day 10.5. While deletion of only Patched1 as well as Patched1 together with RBP-J leads to formation of medulloblastoma concomitant with disorganisation of cell layers, loss of RBP-J from granule neuron progenitors has no obvious effect on overall cerebellar morphology or differentiation and maturation of the different cerebellar cell types.

**Conclusions:**

Our results suggest that even though Notch signalling has been shown to play important roles in cerebellar development, signalling via RBP-J is surprisingly not required in granule neuron progenitors. Furthermore, RBP-J inactivation in these cells does not influence the formation of medulloblastoma initiated by Hedgehog pathway activation. This may suggest a requirement of Notch in cerebellar development at a different developmental stage or in a different cell type than examined here - for example, in the neural stem cells of the ventricular zone. In addition, it remains a possibility that, in granule neuron progenitors, Notch may signal via an alternative pathway without the requirement for RBP-J.

## Background

The Notch signalling pathway plays crucial roles in brain development and in the cerebellum in particular. The main function of Notch is preventing (premature) differentiation of neural progenitor cells and at later stages of neural development promoting glial over neuronal cell fates [1-5]. *In vitro *analyses as well as *in vivo *murine models have shown that, in the cerebellum, the Notch pathway influences the development of Bergmann glia and the differentiation of granule neuron progenitors (GNPs) [6-10].

There are four Notch receptor paralogues (Notch1-4) in vertebrates, all of which bind transmembrane ligands of the Delta-like and Jagged family [3,11,12]. Upon binding of ligand, the Notch receptor is proteolytically cleaved and its intracellular domain translocates to the nucleus where it forms a complex with several co-factors and the DNA binding protein RBP-J (Recombination signal binding protein for immunoglobulin kappa J region; also called CSL for **C**BF1 in mammalians, **S**uppressor of Hairless in *Drosophila*, **L**ag-1 in *C. elegans*), the major canonical downstream effector of the Notch pathway [3,13,14]. The complex then facilitates the expression of target genes, including basic helix-loop-helix transcription factors, such as *Hes1 *and *Hes5*, which act as transcriptional repressors of proneural genes [15-18]. Possible alternative 'non-canonical' downstream pathways of Notch that are RBP-J independent have also been reported [19-22].

Of the Notch receptors, Notch1, Notch2 and Notch3 are most important for cerebellar development and the roles of each receptor vary at different developmental stages [7,23]. During granule neuron development, *Notch2 *is expressed predominantly in proliferating GNPs of the external germinal layer (EGL), and *Notch1 *in postmitotic differentiating cells in the internal granule layer (IGL) [10,24]. Of the five Notch ligands, Delta-like 3 (Dll3) is mainly expressed in GNPs while Jagged2 is expressed in differentiating and mature neurons of the IGL [25]. The specific expression patterns of Notch receptors and ligands in cerebellar development suggest diverse and cell-type-specific roles for the Notch pathway; however, detailed functions of Notch components in cerebellar cell types are not known. Accordingly, we examine here the role of canonical Notch signalling in cerebellar GNPs.

In addition to its function in normal brain development, the Notch pathway has also been implicated in tumour formation, in particular in medulloblastoma. Notch can act as an oncogene or tumour suppressor and different receptors appear to have varying tumorigenicity [24]. In various mouse models of medulloblastoma initiated by perturbation of the Hedgehog (Hh) pathway, Notch pathway components are upregulated, indicating both a potentially oncogenic role for Notch and possibly an interaction between the two pathways [26,27]. Hh signalling controls many patterning events of embryonic development via cell cycle regulation, and deregulation of the pathway often results in tumorigenesis. In the absence of Hh ligand the transmembrane receptor Patched1 (Ptc1) inhibits Smoothened (Smo) while Hh binding results in a release of the repression of Smo by Ptc1 and in a cascade of phosphorylation events [28,29]. Consequently, active Gli transcription factors translocate to the nucleus where they can activate expression of target genes such as *Cyclins D1*, *D2 *and *E *and *N-Myc *[30-34].

We have shown recently that the Hh and Notch pathways interact during cerebellar development in ventricular zone stem cells but this interaction remains to be defined at other stages of cerebellar development and in other cerebellar cell types [35]. In particular, even though Notch pathway components are clearly expressed in the EGL of the developing cerebellum at a time when GNPs are dividing rapidly and then differentiating under the influence of Purkinje-neuron-derived Sonic hedgehog (Shh), it is not known whether Notch signalling is actually required for appropriate formation of granule neurons.

We addressed the interaction of Notch and Hh signalling during granule neuron development and in medulloblastoma formed from GNPs by adopting a genetic approach *in vivo *to ablate Notch signalling in the Ptc1 conditional mouse model, which is a widely accepted and relevant model for human medulloblastoma [36-38]. Since the developing EGL expresses multiple components of the Notch signalling pathway, we took the approach to inactivate the common pathway effector RBP-J in order to ablate canonical Notch receptor signalling. This strategy has been previously applied in analyses of Notch signalling and has proved to be highly effective [8,35,39,40]. A Cre-expressing transgenic line facilitated deletion of the two genes in Math1+ cells (Math1-Cre) of the rhombic lip and EGL [38]. Surprisingly, we found no requirement for RBP-J for the proliferation, differentiation or migration of granule neuron precursors. Also, loss of RBP-J concomitant with activation of the Hh pathway in GNPs did not block medulloblastoma formation. Our data suggest that the function of Notch signalling, as distinct from the expression of Notch signalling components during cerebellar development, is restricted to cell types other than GNPs, that is, stem cells of the ventricular zone and cerebellar cells originating from there.

## Results

### Math1-Cre efficiently inactivates the *Patched1 *and *RBP-J *genes in the external germinal layer

**Figure 1:**
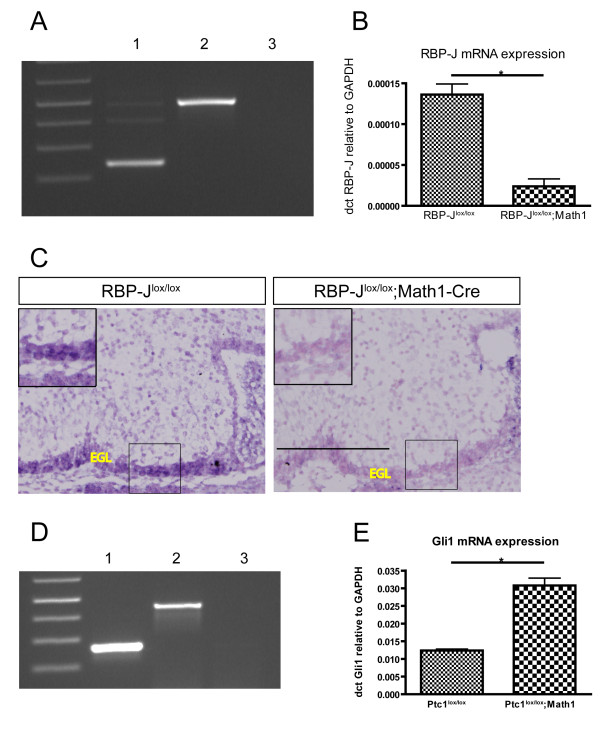
**Math1-Cre deletes the conditional *RBP-J *and *Patched1 *alleles with high efficiency**. **(A) **Floxing analysis of the *RBP-J *allele by RT-PCR with primers flanking the excised region. Samples were isolated GNPs of P7 RBP-J^lox/lox ^mice (pool of 4) and RBP-J^lox/lox^;Math1-Cre mice (pool of 3). Bands resulting from RT-PCR show high efficiency of floxing in mutants (lane 1, 250 bp) compared to controls (lane 2, 498 bp). Lane 3 shows the negative control for the PCR. **(B) **quantitative real-time PCR shows decreased *RBP-J *mRNA levels (relative to *GAPDH*) in RBP-J^lox/lox^;Math1-Cre mice (pool of 3) compared to RBP-J^lox/lox ^mice (pool of 4) (statistical analysis on means of three technical replicates for two aliquots of each cDNA pool, *P *= 0.0194). **(C) ***in situ *hybridisation shows loss of *RBP-J *mRNA in the EGL of E18.5 RBP-J^lox/lox^;Math1-Cre embryos. Images were taken at the same magnification; scale bar represents 200 μm. **(D) **Floxing analysis of the *Ptc1 *allele by RT-PCR with primers flanking the excised region. Samples were isolated GNPs of P7 Ptc1^lox/lox ^mice (pool of 4) and Ptc1^lox/lox^;Math1-Cre mice (pool of 3). Bands resulting from RT-PCR confirm high efficiency of floxing in mutants (lane 1, 250 bp) compared to controls (lane 2, 450 bp). Lane 3 shows the negative control for the PCR. **(E) **Quantitative real-time PCR indicates increased *Gli1 *mRNA levels (relative to *GAPDH*) in Ptc1^lox/lox^;Math1-Cre mice (pool of 3) compared to Ptc1^lox/lox ^mice (pool of 4), supporting activation of the Hh pathway by *Ptc1 *deletion (statistical analysis on means of three technical replicates for two aliquots of each cDNA pool, *P *= 0.0132).

**Figure 2:**
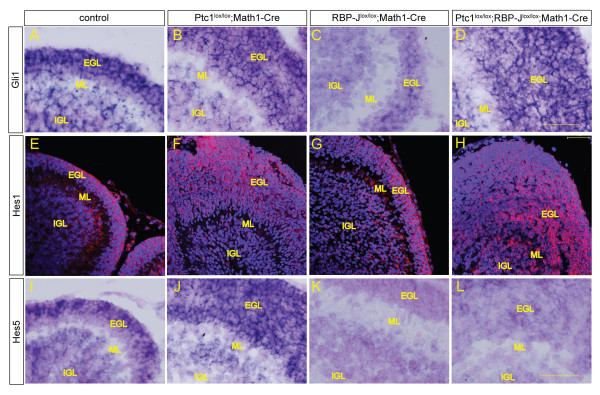
**Deletion of *Patched1 *and *RBP-J *in granule neuron progenitors results in the expression of Hedgehog pathway target genes and loss of Notch pathway target genes**. **(A-D) **The universal Hedgehog target *Gli1 *is expressed in control GNPs as well as in the EGL of all mutants at postnatal day 7, as shown by *in situ *analysis. **(E-H) **Immunofluorescence staining of P7 brain tissue shows the canonical Notch target Hes1 is expressed in the outer EGL in RBP-J deleted GNPs (G), comparable to controls (E) and the number of Hes1 expressing cells in the EGL of *Ptc1 *deleted (F) and *Ptc1 *and *RBP-J *deleted brains is increased (H). **(I,J) **Hedgehog activation in GNPs slightly increases the area of *Hes5 *mRNA expression (J) compared to controls (I). **(K,L) ***In situ *hybridisation for the canonical Notch target Hes5 confirms loss by deletion of *RBP-J *(K) in P7 brains and loss of *Hes5 *in most but not all GNPs of Ptc1^lox/lox^;RBP-J^lox/lox^;Math1-Cre tumours (L). Images in each panel were taken at the same magnification. Counter stain for *in situ *hybridisation was nuclear fast red and for immunofluorencence DAPI. Scale bars represent 50 μm for all images. ML, molecular layer.

Math1-Cre results in Cre expression in Math1-positive cells of the rhombic lip from embryonic day 10.5 (E10.5), and in GNPs in the EGL that have not begun to differentiate into mature neurons [38]. To validate the model used in this study, we first confirmed deletion of both the *Patched1 *and *RBP-J *alleles by Math1-Cre. GNPs isolated from P7 RBP-J^lox/lox^; Math1-Cre mice show high efficiency of floxing by RT-PCR with primers flanking the excised region (exons 6 and 7; Figure 1A). Floxing of *RBP-J *was confirmed by Taqman and *in situ *analysis for *RBP-J *mRNA, which showed significant loss of wild-type *RBP-J *mRNA in mutant GNPs compared to controls (Figure 1B,C). In addition, we assayed mRNA expression of the direct Notch target gene *Hey2 *by quantitative real time PCR (Additional file 1). *Hey2 *mRNA is significantly decreased after *RBP-J *deletion (*P *= 0.0157). The *Patched1 *allele was also deleted with high efficiency by Math1-Cre, as shown by RT-PCR with primers flanking the excised exon 3. Pathway activation due to *Patched1 *deletion was confirmed by Taqman analysis showing significant upregulation of the universal Hh target *Gli1 *in Ptc1^lox/lox^; Math1-Cre GNPs compared to Ptc1^lox/lox ^GNPs (Figure 1D,E). To further validate our model, we performed *in situ *hybridisation and immunofluorescence staining to characterise the expression of Hh and Notch pathway targets in the mutants. *In situ *analysis for *Gli1 *supports Hh activation in the EGL of *Ptc1 *deleted as well as *Ptc1 *and *RBP-J *deleted mice (Figure 2B,D). Interestingly, our *in situ *analysis also suggests a reduction of *Gli1 *expression in RBP-J^lox/lox^; Math1-Cre cerebella, indicating that *RBP-J *deleted GNPs might not be able to adequately respond to Shh ligand secreted by Purkinje neurons (Figure 2C). The canonical Notch target *Hes1 *(Hairy enhancer of split) is expressed in the outer EGL of control mice (Figure 2E). However, *Hes1 *has also been reported to be a target of Hh signalling [41,42] and the Hes1-positive area appears expanded in response to Hh activation, with staining throughout the EGL of *Ptc1 *deleted brains (Figure 2F). RBP-J deletion neither leads to loss of Hes1 nor can it overcome the increase of Hes1-positive cells in the *Ptc1 *deleted cerebellum (Figure 2G,H). The canonical Notch target *Hes5 *is expressed in the outer EGL of control mice, with an expansion towards the inner EGL in Hh activated mice, similar to Hes1 (Figure 2I,J). *RBP-J *deletion results in loss of *Hes5 *expression; however, some cells of Ptc1^lox/lox^;RBP-J^lox/lox^;Math1-Cre EGL express *Hes5*, possibly due to proliferation of a small number of GNPs with incomplete deletion of *RBP-J *in these mutants (Figure 2K,L). Taken together, our data confirm that Math1-Cre efficiently deletes both *Patched1 *and *RBP-J *alleles and consequently leads to GNP-specific activation of Hh and inactivation of Notch signalling, respectively.

### Loss of *RBP-J *in GNPs does not alter cerebellar morphology

**Figure 3:**
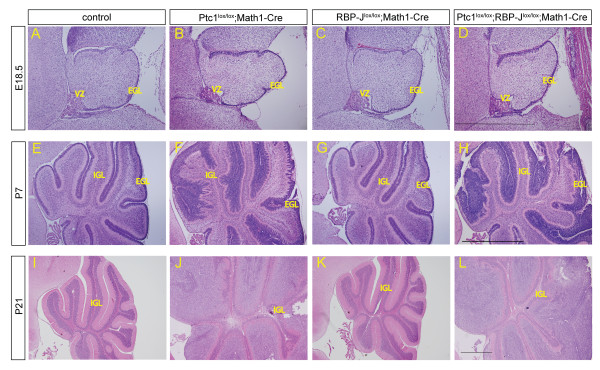
***RBP-J *deletion in granule neuron progenitors does not change cerebellar morphology**. **(A-L) **Haematoxylin and eosin staining of brain sections at E18.5 **(A-D)**, P7 **(E-H)** and P21 **(I-L)**. *Ptc1 *deletion results in progressive thickening of the EGL and medulloblastoma formation by P21 **(B,F,J)**, which is not ameliorated by *RBP-J *deletion **(D,H,L)**. Cerebella of RBP-J^lox/lox^;Math1-Cre mice **(C,G,K)** are undistinguishable from controls **(A,E,I)**. Images in each panel were taken at the same magnification; scale bars represent 1 mm.

To examine the effects of Hh pathway activation and Notch inactivation on the development and differentiation of cerebellar cell types, we first analysed mutant brains at three developmental stages (E18.5, postnatal day 7 (P7) and P21) histologically by haematoxylin and eosin staining. At E18.5 all mutants are morphologically similar to control cerebellum, with a normal appearing EGL and beginning foliation (Figure 3A-D). By P7, control cerebellum has developed ten folia and GNPs have begun to differentiate and form an IGL, which is fully developed by P21 with no EGL remaining (Figure 3E,I). *RBP-J *deletion has no discernable effect on morphology throughout cerebellar development (Figure 3G,K). *Ptc1 *deletion results in an increase of GNPs in the EGL and, therefore, a thickening of this region, which develops into medulloblastoma by P21. Foliation appears largely unaffected, with the correct number of lobes present, and some GNPs have been able to differentiate and migrate to form an IGL. The boundaries between the EGL, molecular layer (ML) and IGL appear disturbed, likely due to the excessive expansion of GNPs (Figure 3F,J). Notch inhibition by *RBP-J *deletion does not alter this phenotype (Figure 3H,L). In summary, GNP-specific deletion of *RBP-J *does not affect the morphology of the cerebellum with or without *Ptc1 *deletion.

### *RBP-J *deletion does not influence the development of Hedgehog-pathway-induced medulloblastoma

**Figure 4:**
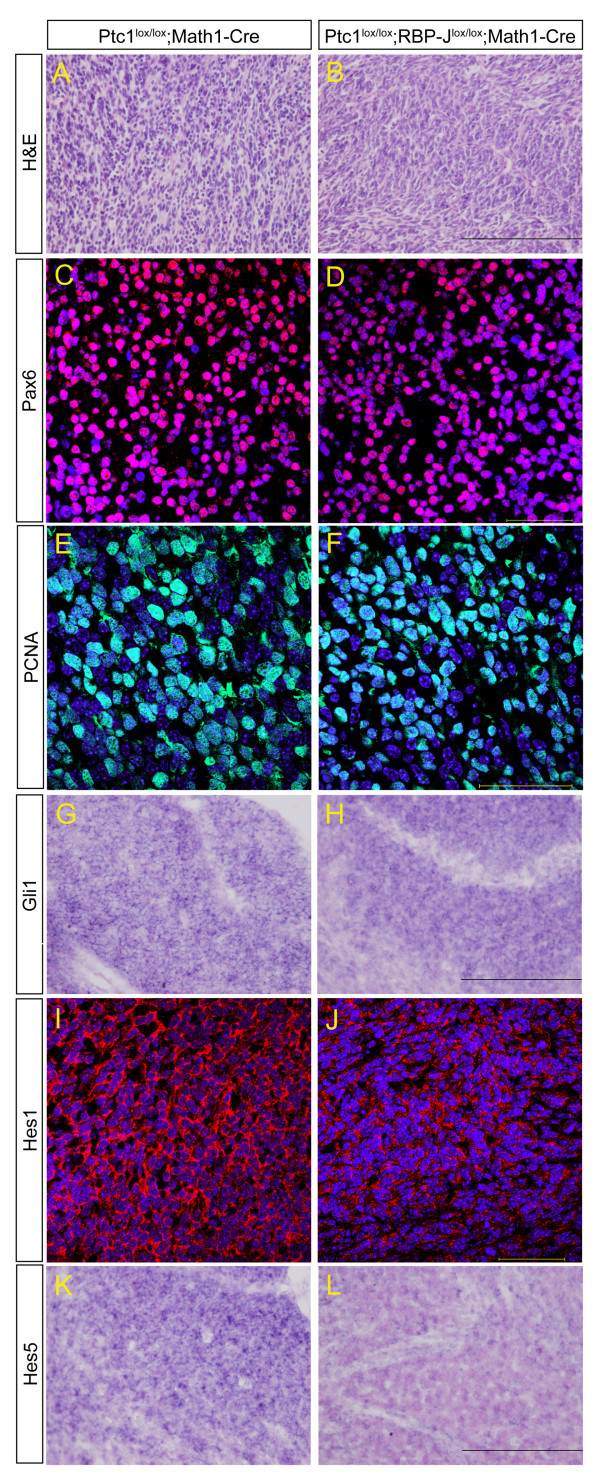
***RBP-J *deletion in granule neuron progenitors does not influence the formation of Hedgehog-pathway-dependent medulloblastoma**. **(A-L) **Cerebellar tumours of Ptc1^lox/lox^;RBP-J^lox/lox^;Math1-Cre mice (B,D,F,H,J,L) closely resemble those of Ptc1^lox/lox^;Math1-Cre mice (A,C,E,G,I,K). Comparison was based on overall morphology by haematoxylin and eosin staining (A,B), granule cell identity by immunofluorescence staining for Pax6 (C,D), proliferation by immunofluorescence staining for proliferating cell nuclear antigen (PCNA) (E,F) and Hh pathway activity by *in situ *hybridisation for *Gli1 *(G,H). The canonical Notch target Hes1 is expressed in tumours of both genotypes as assayed by immunofluorescence staining as it can also be upregulated in response to Hh signals (I,J). Hes5, however, which even when upregulated as a response to Hh signalling, requires RBP-J, is lost in most cells of Ptc1^lox/lox^;RBP-J^lox/lox^;Math1-Cre tumours (K) while being strongly expressed in all cells of Ptc1^lox/lox^;Math1-Cre tumours (L). Both images in each pair were taken at the same magnification; scale bars represent 200 μm for haematoxylin and eosin, Gli1 and Hes5, and 50 μm for Pax6, Hes1 and PCNA staining.

Mice with GNP-specific deletion of the negative Hh regulator Ptc1 develop severe medulloblastoma by P21 in 100% of cases and this phenotype, as well as the overall morphology (Figure 3J,L) are not altered by loss of canonical Notch signalling. We examined tumours of both genotypes (Ptc1^lox/lox^;Math1-Cre and Ptc1^lox/lox^;RBP-J^lox/lox^;Math1-Cre) in more detail and observed that the tumour mass is made up of small round granular cells interspersed with stroma in both cases (Figure 4A,B), similar to human medulloblastoma and as previously published [38]. Both also stain positively for the granule cell marker Pax6, confirming a GNP origin of the tumours (Figure 4C,D). Staining with proliferating cell nuclear antigen (PCNA) reveals that most cells in both genotypes are actively undergoing proliferation (Figure 4E,F). The universal Hh target *Gli1 *is expressed in *Ptc1 *deleted tumours as well as in those with additional loss of *RBP-J *(Figure 4G,H), as is *Hes1*, a target of both the Hh and Notch pathways (Figure 4I,J). The canonical Notch target *Hes5*, however, which requires RBP-J, is expressed in Hh activated tumours but lost in most cells of tumours with activated Hh and inactivated Notch signalling (Figure 4K,L). In conclusion, even though Notch signalling is lost in the majority of cells in Ptc1^lox/lox^;RBP-J^lox/lox^;Math1-Cre tumours, as confirmed by loss of *Hes5 *mRNA, *RBP-J *deletion does not appear to influence the characteristics of medulloblastoma initiated by *Ptc1 *deletion in GNPs.

### Differentiation of cerebellar cell types does not depend on canonical Notch signalling via RBP-J

**Figure 5:**
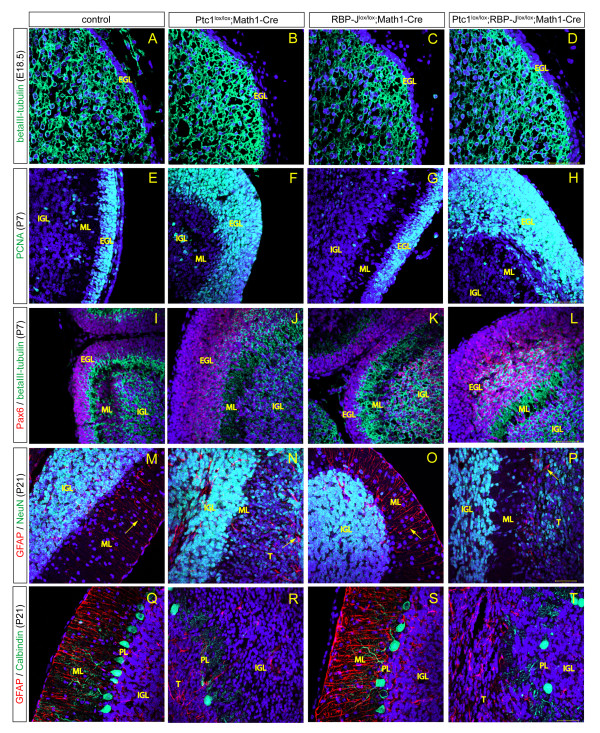
***RBP-J *deletion does not affect the differentiation of cerebellar cell types**. **(A-D) **At E18.5 neuronal differentiation is occurring normally in all mutants as shown by similar staining for betaIII-tubulin. **(E,G) **In P7 cerebella of controls (E) and Notch-inactivated mutants (G), cells of the outer EGL are proliferating and stain positive for PCNA. **(F,H) **In Ptc1^lox/lox^; Math1-Cre and Ptc1^lox/lox^; RBP-J^lox/lox^; Math1-Cre cerebella, proliferating cells populate the whole EGL, including the inner region. **(J,L) **Pax6, a marker for granule neurons, indicates differentiation is initiated in *Ptc1 *deleted brains (J) and Ptc1^lox/lox^;RBP-J^lox/lox^;Math1-Cre cerebella (L). **(I,K) ***RBP-J *deleted cerebella (K) show normal differentiation of GNPs compared to controls (I) at P7. **(M-P) **At P21 mature neurons in the EGL express NeuN while Bergmann glia express glial fibrillary acidic protein (GFAP). Bergmann glial fibres are aligned in parallel, spanning the ML (arrows). In *RBP-J *deleted cerebella (O) neither neuronal nor glial development appears impaired compared to controls (M). *Ptc1 *deleted cerebella have an IGL with NeuN-positive differentiated neurons; however, NeuN-positive cells are also present interspersed in the expanded EGL (T, tumour). In addition, Bergmann glia in Hh-activated cerebella appear disorganised without parallel alignment of their fibres (N). The effect of *Ptc1 *deletion on neurons and Bergmann glia cannot be overcome by Notch inactivation (P). **(Q,S) **Purkinje neurons marked by Calbindin are organised in a single layer between the ML and IGL of control (Q) as well as *RBP-J *deleted cerebella (S). **(R,T) **Hh activation results in disorganisation of the Purkinje cell layer (PL) (R), which is not changed by additional RBP-J deletion (T). Images in each panel were taken at the same magnification and nuclei were counterstained with DAPI. Scale bars represent 50 μm.

Following the characterisation of *Ptc1 *and *RBP-J *deleted medulloblastoma, we investigated cerebellar development in more detail by using immunofluorescence staining with markers specific for different cerebellar cell types. At E18.5, GNPs have migrated from the rhombic lip to form the EGL and neuronal differentiation is in progress. BetaIII-tubulin staining confirmed the presence of immature neurons migrating away from the EGL in control embryos and in all mutants (Figure 5A-D). GNPs in the EGL proliferate in response to Shh, which is secreted by Purkinje neurons, and at P7 the peak of proliferation is reached. PCNA staining confirmed that cells in the outer EGL of the control and *RBP-J *deleted cerebellum are mitotically active (Figure 5E,G). *Ptc1 *deletion results in an expansion of the proliferative compartment, which is not influenced by loss of Notch signalling (Figure 5F,H). However, even when *Ptc1 *deletion is present, GNPs in the inner EGL can differentiate and begin to express betaIII-tubulin in addition to the granule cell marker Pax6 (Figure 5J). The same is evident when the Notch pathway is inhibited in addition to Hh activation (Figure 5L) and differentiation of RBP-J^lox/lox^;Math1-Cre GNPs into neurons is initiated normally (Figure 5K,I). GNPs isolated from P7 to P8 cerebellum and stained for PCNA, betaIII-tubulin, NeuN (Neuronal nuclei) and by TUNEL *in vitro *confirm no obvious difference in proliferation, differentiation and apoptosis rates between RBP-J^lox/lox ^controls and RBP-J^lox/lox^;Math1-Cre mutants (Additional file 2A-H). Culture of GNPs for 3 days in the absence and presence of recombinant Shh-N reveals that *RBP-J *deletion also has no influence on the proliferative response to Shh as assayed by immunofluorescence for PCNA (Additional file 2I-L) and cell count (Additional file 2M). By P21 most cerebellar differentiation processes are complete and the layering of the mature cerebellum is present. Mature, NeuN-positive neurons populate the IGL while Bergman glial fibres, marked by glial fibrillary acidic protein (GFAP), span the ML in parallel to each other (Figure 5M). Notch ablation does not influence either of these cell types (Figure 5O). *Ptc1 *deletion, however, results in a severe disorganisation of Bergman glial fibres as well as the presence of NeuN-positive cells in the remainder of the expanded EGL. These cells are likely to be differentiated granule neurons that were hindered from migrating into the IGL by the bulk of proliferating tumour cells (Figure 5N). The same phenotype is evident in Hh-activated cerebella with additional *RBP-J *deletion (Figure 5P). Purkinje neurons, marked by Calbindin, are organised in a single layer around the surface of the IGL with their fibres reaching into the ML in control brains as well as in Notch deficient cerebella (Figure 5Q,S). In Hh-activated as well as Hh-activated and Notch-inactivated brains the Purkinje neuron layer is disrupted and lacks organisation of cell bodies as well as orientation of their fibres (Figure 5R,T). Overall, the differentiation of cerebellar cell types appears unaffected by *RBP-J *deletion and the disorganisation of cell layers in medulloblastoma initiated by *Ptc1 *deletion cannot be overcome by canonical Notch inactivation.

### GNP-specific deletion of *RBP-J *and *Ptc1 *does not influence the cerebellar stem and progenitor cell pool

Next we asked whether Hh activation and/or Notch inactivation in GNPs influences the stem and progenitor cell pool of developing cerebella. To examine cerebellar stem cell properties, we used Sox2, which marks neural stem cells and Bergman glia. In controls as well as all mutants, cerebellar stem cells residing in the ventricular zone (VZ) stain positive for Sox2. In addition, all genotypes show some Sox2-positive cells throughout the cerebellum, Bergman glia originating from the VZ, which are migrating towards the ML (Additional file 3A-D). In Ptc1^lox/lox^; Math1-Cre mice background staining appears increased, although there is no apparent difference in nuclear Sox2 staining (Additional file 3B). Furthermore, we utilised the neurosphere assay, a widely used tool to examine stem/progenitor cell numbers. The number of colonies counted in this assay is indicative of the number of stem/progenitor cells in the population but it cannot distinguish between the two. No significant difference was observed in colony numbers after deletion of *Ptc1 *or *RBP-J *compared to controls after 5 days of incubation of single cell suspensions of P7 cerebella (Additional file 3E). Taken together, GNP-specific deletion of *Ptc1 *and *RBP-J *appears to have no effect on the cerebellar stem/progenitor cell pool.

## Discussion

The Notch signalling pathway influences cerebellar development, in particular the differentiation of neurons and glia. Loss of the Notch1 receptor or the ligand Jagged1 in neuroepithelial cells results in premature differentiation of GNPs and defects in neuronal migration, and RBP-J plays a crucial role in the development and migration of Bergman glia [8,43,44]. Signalling from the Notch2 receptor appears to have a role opposing that of Notch1 in GNPs, promoting proliferation while inhibiting differentiation [10,24]. Several studies have also suggested a role for Notch signalling in the formation of medulloblastoma [26,27,45]. Multiple components of the Notch pathway are expressed in the EGL, so here we investigate the role of canonical Notch signalling in GNPs by deletion of the common Notch effector RBP-J in Math1+ cells and the consequences of Notch signal inactivation on the initiation and development of Hh-pathway-dependent medulloblastoma.

First we confirmed the validity of our model using RT-PCR, quantitative real-time PCR and *in situ *analysis, showing high efficiency of floxing for both the *Ptc1 *and *RBP-J *alleles, resulting in Hh pathway upregulation and Notch inactivation in GNPs, respectively (Figures 1 and 2; Additional file 1). The overall morphology of cerebella with *RBP-J *deleted GNPs appeared normal, with foliation and layer formation identical to control cerebella. Hh activation by *Ptc1 *deletion had no effect until after birth, when GNPs in the EGL excessively proliferate and lead to medulloblastoma formation by P21 in all individuals, as we have shown previously [38] (Figure 3). Even though *RBP-J *deletion did not appear to alter overall cerebellar development, we then asked if it had a more subtle impact on differentiation or migration of cerebellar cell lineages. Notch signalling has been shown to be critical for neuronal and glial differentiation and migration in cerebellar development and we therefore examined the different cell types of the cerebellum using lineage-specific markers. We found that loss of *RBP-J *from GNPs has no effect on neuronal differentiation and migration. This finding appears to be in direct contrast to a report demonstrating defects in granule cell migration after loss of the Notch ligand Jagged1 [44]. However, deletion in the above-mentioned study led to a deficit of Bergman glia, which act as migratory scaffolds for GNPs, and it was therefore found that the GNP defect was likely secondary to the loss of Bergmann glia. Furthermore, GNP-specific *RBP-J *deletion cannot overcome the cerebellar disorganisation resulting from *Ptc1 *deletion and medulloblastoma formation (Figures 4 and 5). This raises the question of whether Notch signalling in GNPs is transduced via alternative pathways that are independent of canonical Notch signalling via RBP-J. Indeed, work by Mizutani *et al*. [46] indicates that, in contrast to neural stem cells, more committed neural progenitor cells may use alternative Notch pathways without a requirement for RBP-J. In the absence of Notch signals RBP-J could potentially function as a transcriptional repressor, so loss of RBP-J may have an effect even on cells that do not usually require active Notch signalling. It has been suggested that this function of RBP-J does not play a crucial role in mammals and the absence of a phenotype after *RBP-J *deletion in our model confirms this specifically for GNPs [13]. An alternative explanation for the lack of an effect of *RBP-J *deletion on GNPs is that Notch signalling may be required at an earlier time point, before commitment to the granule cell lineage. In accordance with this, inactivation of Notch signalling at early embryonic time points has a severe impact on cerebellar development and Notch has also been implicated in controlling the balance between symmetric and asymmetric stem cell division in a number of tissues, including the brain [[3,44], and our unpublished results]. In addition, we have shown previously that *RBP-J *deletion in the cerebellar VZ induces an increase in progenitor cell numbers in the niche due to a loss of stem cells and a delay in differentiation [35]. Therefore, we next examined the properties of the cerebellar stem/progenitor cell niche by Sox2 staining and neurosphere assays. Neither approach indicated an effect of *RBP-J *deletion (Additional file 3), likely due to the specificity of Math1-Cre, which deletes in GNPs after they have left the VZ, the cerebellar stem cell niche, and committed to the granule cell lineage.

In addition to *RBP-J *deletion having no impact on the differentiation of cerebellar cell types and the stem/progenitor cell niche, we also found that loss of canonical Notch signalling does not influence the formation of Hh-dependent medulloblastoma (Figures 3H,L, 4, and 5D,H,L,P,T). We confirmed loss of *Hes5 *mRNA in the majority of tumour cells and thereby excluded the possibility that the minority of cells deleted for *Ptc1 *but not for *RBP-J *may have had a growth advantage and populated the tumour mass (Figure 4K,L). The absence of any impact of *RBP-J *deletion on tumour formation was surprising as several studies have noted dysregulation of Notch pathway components in both human and murine medulloblastoma, including the transcription factor targets *Hes1 *and *Hes5*, and the expression of *Hes1 *is associated with poor clinical outcome [24,26,27,45,47]. Medulloblastoma cell lines treated with γ-secretase inhibitors that block Notch receptor endoproteolysis display reduced growth, clonogenicity and tumorigenicity, and γ-secretase inhibitors have been proposed as a chemotherapeutic approach to treating medulloblastoma [27,48,49]. Cerebella of mice with both *Ptc1 *and *RBP-J *deletion appear identical in morphology to those with deletion of *Ptc1 *alone, and expression of cell-type-specific markers and all mutant mice develop severe medulloblastoma by P21. This indicates that canonical Notch signalling is likely not required for the development of medulloblastoma initiated by Hh pathway activation in GNPs. However, we cannot exclude the possibility that there might be some influence of *RBP-J *deletion on tumour latency, and a more extensive study with a large number of mutant mice of both genotypes would be required to address this question.

## Conclusions

We have shown here that canonical Notch signalling via RBP-J is not required in GNPs and Hh-pathway-dependent medulloblastoma. A crucial role for Notch signalling in cerebellar development has been shown previously and we conclude that the involvement of the Notch pathway may be restricted to the stem/progenitor cell niche and loses influence as cells commit to the granule neuron lineage. This is despite the observation that Notch pathway components such as Notch1, Notch2, Notch3, Dll3, Jagged1, Hes1, and Hes5 are expressed in the EGL, underlining the fact that it is important not to confuse detectable expression of a signalling pathway with it necessarily functioning in that tissue. Additional work is required to characterise the role of Notch in cerebellar development, in particular to define the developmental stage(s) when it is required and to identify the utilised downstream effectors.

## Materials and methods

### Mouse models

All work involving mice was performed with approval and according to guidelines of the University of Queensland Animal Ethics Committee. Mouse models used were Ptc1 conditional mice [50] and RBP-J conditional mice [51] crossed with a Math1-Cre line (kindly provided by David Rowitch).

### Isolation and culture of granule neuron progenitors

Cerebellar GNPs were isolated from P7 to P8 pups as described previously [38]. In brief, cerebella were dissected and cells dissociated and triturated, followed by centrifugation through a 35 to 65% percoll gradient (Amersham Biosciences, now GE Healthcare Bio-Sciences Corp., Picataway, NJ, USA) in order to segregate granule neurons from astrocytes. Isolated GNPs were washed and used for subsequent experiments.

Culture of GNPs in the presence or absence of 3 μg/ml Shh-N (R&D Systems, Minneapolis, MN, USA) was performed at a density of 5 × 10^5 ^cells per well in 12-well plates with poly-L-lysine (Sigma Aldrich, St Louis, MO, USA) coated coverslips in NB-B27 media (Neurobasal with 1 mM sodium pyrovate, 2 mM L-glutamine, penicillin/streptomycin, and B27 supplement, all from Invitrogen, Carlsbad, CA, USA).

### RT-PCR and quantitative RT-PCR

RNA was extracted from cells using the RNeasy Mini Kit by QIAgen (Hilden, Germany) according to the manufacturer's manual. Reverse transcription was performed using the Superscript III system by Invitrogen. The following primers were used for PCR detection of the RBP-J floxed allele: forward 5' CATCTCCAAACCCTCCAAAA 3', reverse 5' GTCCAGGAAGCTCCATCGT 3'; and the Patched1 floxed allele: forward 5'-CACCGTAAAGGAGCGTTACCTA-3', reverse 5'-TGGTTGTGGGTCTCCTCATATT-3'. Quantitave PCR was performed with Assays on Demand by Applied Biosystems for RBP-J (Mm00770450_m1), Gli1 (Mm00494645_m1), and Hey2 (Mm00469280_m1) according to the manufacturer's protocol on the ABI Prism 7000 equipment by Applied Biosystems (Austin, TX, USA). Measurements were taken in three technical replicates and data were normalized to the housekeeping gene *GAPDH *(assay ID 4352339E). Statistical analysis was performed using Graphpad Prism 4 (Graphpad Software, La Jolla, CA, USA) for unpaired *t*-tests.

### Immunofluorescence, TUNEL and haematoxylin and eosin staining

Brains were dissected (after cardiac perfusion for P7 and P21 mice) and fixed in 4% paraformaldehyde overnight. Subsequently, samples were either embedded in paraffin or cryoprotected in 30% sucrose followed by embedding in OCT compound. Antigen retrieval of deparaffinised wax tissue sections or defrosted cryosections was performed by boiling in antigen unmasking solution (Vector Laboratories, Burlingame, CA, USA). Sections were blocked in 4% horse serum, 1% bovine serum albumin and 0.2% Triton-X in phosphate-buffered saline prior to primary antibody incubation overnight at 4°C. Slides were incubated with secondary antibodies for 1 h at room temperature. For immunofluorescence, a DAPI counterstain (1:10,000; Sigma Aldrich) was performed prior to mounting with Fluorescence Mounting Media (Dako, Carpentaria, CA, USA). For histological analysis, deparaffinised and rehydrated sections were stained in haematoxylin (Vector Laboratories) and eosin Y (Sigma Aldrich) and mounted in Entellan. Antibodies used were betaIII-tubulin (1:2,000; Promega Corporation, Madison, WI, USA), Sox2 (1:200; R&D Systems), PCNA (1:100; Invitrogen), GFAP (1:500; Dako), NeuN (1:100; Chemicon, Temecula, CA, USA), Calbindin (1:200; Sigma) and Hes1 (1:400; a gift from R Kageyama, Kyoto, Japan) on paraffin sections, and Pax6 (1:300; Covance, Princeton, NJ, USA), together with betaIII-tubulin on frozen sections. Fluorescent secondary antibodies used were anti-rabbit Alexa555 (1:250; Invitrogen), anti-mouse Alexa488 (1:250; Invitrogen) and anti-goat Cy3 (1:250; Abacus ALS Pty Ltd, Brisbane, Australia). For Sox2 staining, the brightness of all images was increased by the same level to make positive staining better visible. Terminal deoxynucleotidyl transferase dUTP nick end labeling (TUNEL) was performed with the In Situ Cell Death Detection Kit (Fluorescein; Roche Diagnostics, Mannheim, Germany) according to the manufacturer's protocol.

### *In situ *hybridisation

*In situ *hybridisation was performed as previously published [30]. In summary, probes were prepared using DIG labelled probe amplification followed by phenol/chloroform extraction and precipitation. Paraffin-embedded sections (6 μm) were treated with 2 μg/ml ProK (Roche Diagnostics) in TE buffer, fixed in 4% paraformaldehyde and acetylated. Hybridisation was performed in hybridisation buffer at 64°C for Gli1 and 65°C for Hes5 and RBP-J over night. A series of saline-sodium citrate (SSC) washes was followed by blocking and washing (DIG block and wash buffer set, Roche) before incubation with anti-DIG-AP antibody (Roche). Colour reaction was performed using 3.5 mg/ml nitroblue tetrazolium (NBT) and 1.75 mg/ml 5-bromo-4-chloro-3-indolyl-phosphate (BCIP) (both Roche) in 10% polyvinylalcohol (PVA) (Sigma). Colour formation was stopped in TE solution followed by counter staining with nuclear fast red (Vector Labs), post-fixation and mounting.

Probes used were Gli1 (a gift from A Joyner, New York, NY, USA), RBP-J (a gift from T Honjo, Kyoto, Japan) and Hes5 (a gift from R Kageyama, Kyoto, Japan).

### Microscopy

Light and general fluorescence microscopy were performed using an Olympus BX-51 upright microscope. Confocal images were taken on a Zeiss LSM 510 META.

### Stem/progenitor cell analysis

P7 cerebellar cells were harvested and subsequently dissociated. For neurosphere assays, cells were plated at a density of 1 × 10^5 ^cells per ml in 200 μl Neurosphere assay media (Neurosphere media containing 10% Neurocult neural stem cell proliferation supplement (Stem Cell Technologies, Tullamarine, VIC), 5% bovine serum albumin (Sigma), 1% penicillin/streptomycin) containing epidermal growth factor (20 ng/ml) in a 96-well plate. For each individual the assay was set up in triplicates. The number of spheres per well was counted 5 days after plating. Statistical analysis was performed using Graphpad Prism 4 for unpaired *t*-tests.

## Abbreviations

E: embryonic day; EGL: external germinal layer; GFAP: glial fibrillary acidic protein; GNP: granule neuron progenitor; Hes1: Hairy enhancer of split 1; Hh: Hedgehog; IGL: internal granule layer; ML: molecular layer; NeuN: Neuronal nuclei; P: postnatal day; PCNA: proliferating cell nuclear antigen; Ptc1: Patched1; RBP-J: Recombination signal binding protein for immunoglobulin kappa J region; Shh: Sonic hedgehog; TUNEL: terminal deoxynucleotidyl transferase dUTP nick end labelling; VZ: ventricular zone

## Competing interests

The authors declare that they have no competing financial interests or other conflicts in relation to the work described in this paper.

## Authors' contributions

EJ carried out all experimental work and drafted the manuscript. ARH participated in data analysis and critical reading of the manuscript. BJW conceived of the study, and participated in data analysis and in drafting of the manuscript.

## Supplementary Material

Additional file 1 **Figure S1 - *RBP-J *deletion in granule neuron precursors leads to loss of Notch target gene expression**. Quantitative realtime PCR shows decreased mRNA expression of the direct Notch target *Hey2 *in RBP-J^lox/lox^;Math1-Cre (pool of 3) compared to RBP-J^lox/lox ^(pool of 4) granule neuron progenitors (statistical analysis on means of three technical replicates for two aliquots of each cDNA pool, *P *= 0.0157). Click here for file

Additional file 2 **Figure S2 - *RBP-J *deletion does not alter the properties of GNPs *in vitro***. **(A-F) **Granule neuron progenitors isolated from 7- to 8-day-old RBP-J^lox/lox ^(pool of 4) and RBP-J^lox/lox^; Math1-Cre (pool of 5) cerebella show no difference in staining for PCNA (A,B) or the neuronal markers betaIII-tubulin (C,D) and NeuN (E,F). **(G,H) **TUNEL staining also reveals comparable rates of apoptosis in cells of both genotypes. **(I-M) **Culture of GNPs in the presence of 3 μg/ml Shh results in a modest increase in cells stained positively for PCNA in both genotypes (I,J) compared to untreated cells (K,L); however, the number of cells counted per well is not significantly different (M). Examples of positively stained cells are highlighted by arrows. Nuclei were counterstained with DAPI. Scale bars represent 50 μm. Click here for file

Additional file 3 **Figure S3 - *RBP-J *deletion in granule neuron progenitors does not influence the cerebellar stem cell pool**. Neuronal stem cells that reside in the VZ are unaffected by deletion of *Ptc1 *or *RBP-J *as shown by Sox2, a marker of neural stem cells and Bergmann glia. **(A-D) **Sox2 staining appears similar in all genotypes at E18.5, with positive cells in the VZ (magnified insets) and some Sox2-expressing Bergman glia migrating towards the ML, apart from a slight increase in background staining in *Ptc1 *deleted cerebellum. Nuclei were counterstained with DAPI. Scale bar represents 100 μm. **(E) **Neurosphere assays of cells isolated from P7 cerebellum also show no significant difference between controls and *Ptc1 *or *RBP-J *deleted mutants. For statistical analysis, unpaired *t*-tests were performed using Graphpad Prism 4. Click here for file
