# Supplementary figures and images for: RBP-J is not required for granule neuron progenitor development and medulloblastoma initiated by Hedgehog pathway activation in the external germinal layer
Source: Neural Dev. 2010 Oct 15;5:27. doi: 10.1186/1749-8104-5-27 (PMC2972267; doi:10.1186/1749-8104-5-27)

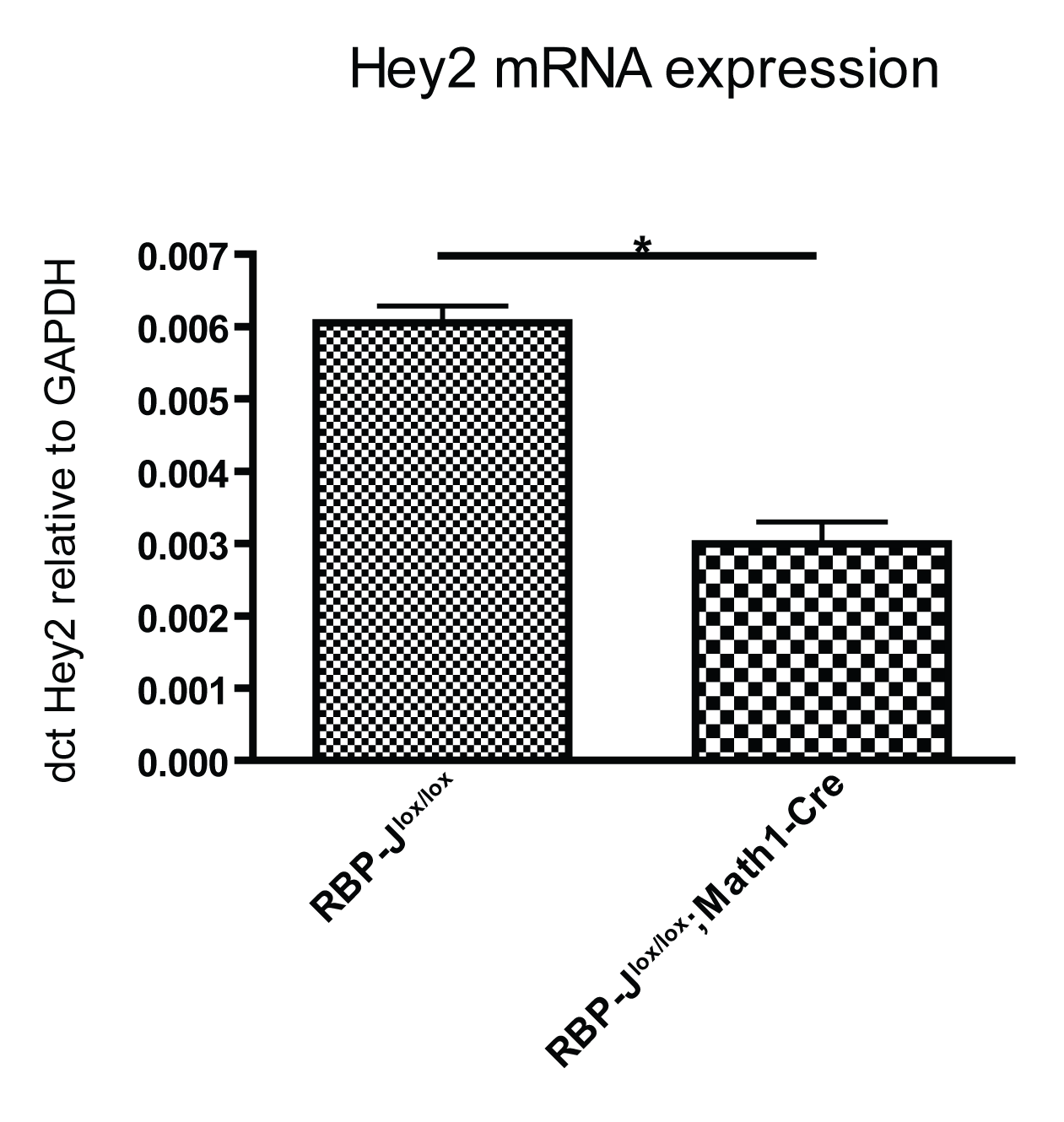

Supplement: Additional file 1 — Figure S1 - RBP-J deletion in granule neuron precursors leads to loss of Notch target gene expression. Quantitative realtime PCR shows decreased mRNA expression of the direct Notch target Hey2 in RBP-Jlox/lox;Math1-Cre (pool of 3) compared to RBP-Jlox/lox (pool of 4) granule neuron progenitors (statistical analysis on means of three technical replicates for two aliquots of each cDNA pool, P = 0.0157). [file 1749-8104-5-27-S1.tiff]

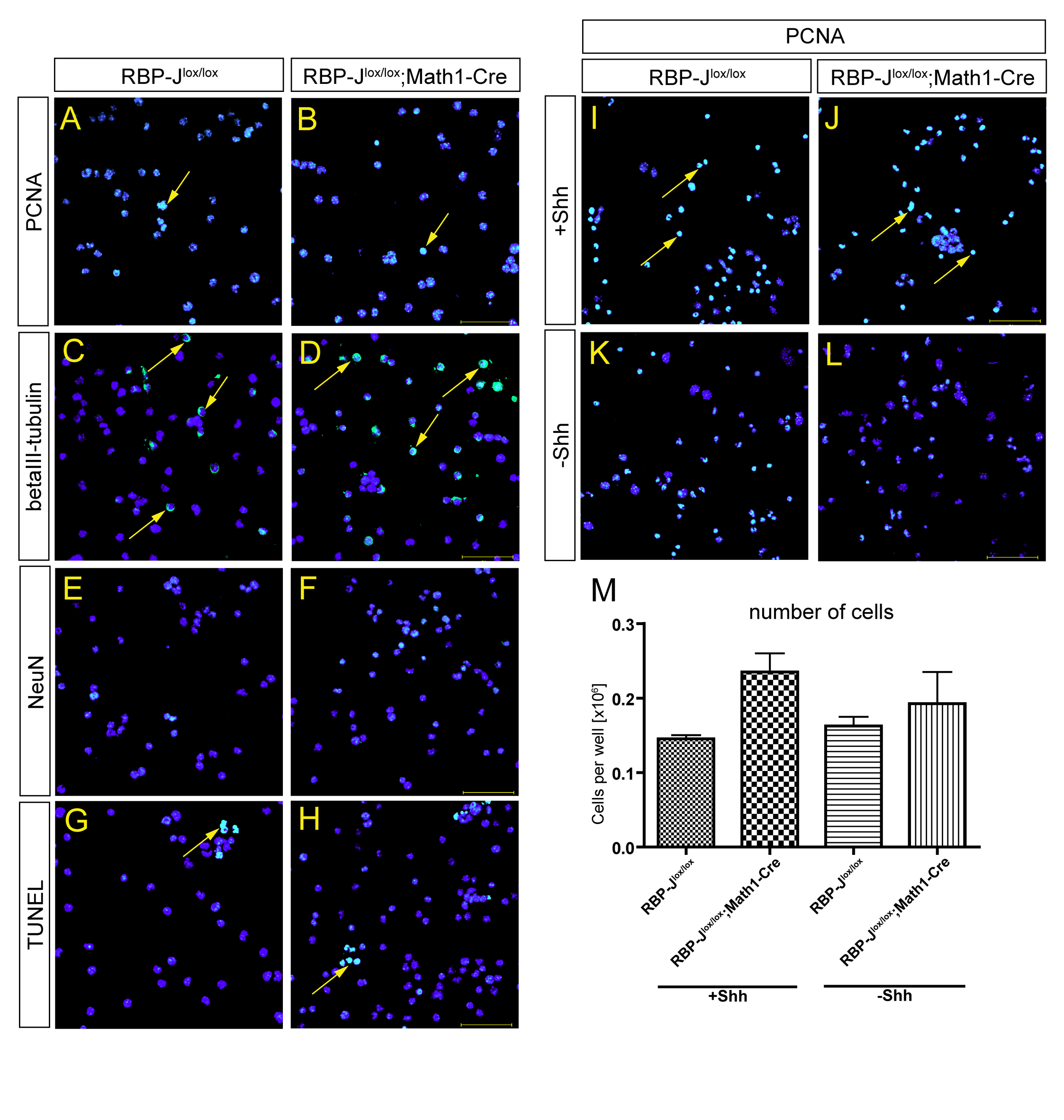

Supplement: Additional file 2 — Figure S2 - RBP-J deletion does not alter the properties of GNPs in vitro. (A-F) Granule neuron progenitors isolated from 7- to 8-day-old RBP-Jlox/lox (pool of 4) and RBP-Jlox/lox; Math1-Cre (pool of 5) cerebella show no difference in staining for PCNA (A,B) or the neuronal markers betaIII-tubulin (C,D) and NeuN (E,F). (G,H) TUNEL staining also reveals comparable rates of apoptosis in cells of both genotypes. (I-M) Culture of GNPs in the presence of 3 μg/ml Shh results in a modest increase in cells stained positively for PCNA in both genotypes (I,J) compared to untreated cells (K,L); however, the number of cells counted per well is not significantly different (M). Examples of positively stained cells are highlighted by arrows. Nuclei were counterstained with DAPI. Scale bars represent 50 μm. [file 1749-8104-5-27-S2.tiff]

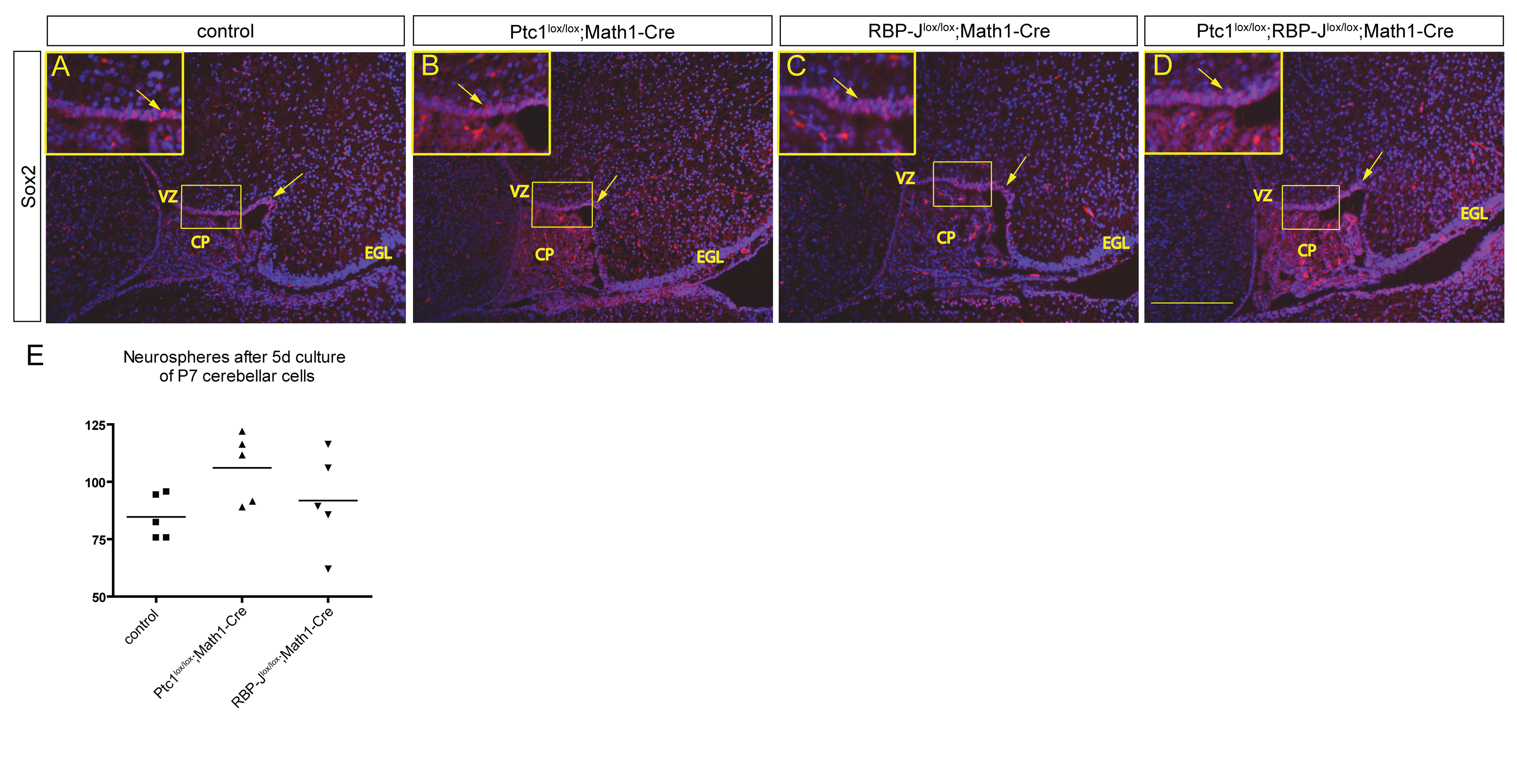

Supplement: Additional file 3 — Figure S3 - RBP-J deletion in granule neuron progenitors does not influence the cerebellar stem cell pool. Neuronal stem cells that reside in the VZ are unaffected by deletion of Ptc1 or RBP-J as shown by Sox2, a marker of neural stem cells and Bergmann glia. (A-D) Sox2 staining appears similar in all genotypes at E18.5, with positive cells in the VZ (magnified insets) and some Sox2-expressing Bergman glia migrating towards the ML, apart from a slight increase in background staining in Ptc1 deleted cerebellum. Nuclei were counterstained with DAPI. Scale bar represents 100 μm. (E) Neurosphere assays of cells isolated from P7 cerebellum also show no significant difference between controls and Ptc1 or RBP-J deleted mutants. For statistical analysis, unpaired t-tests were performed using Graphpad Prism 4. [file 1749-8104-5-27-S3.tiff]
